# Orthopaedic training for non-orthopaedic providers: A review

**DOI:** 10.6026/973206300212543

**Published:** 2025-08-31

**Authors:** Prajakta Warjukar, Ankush Mohabey, Sitanshu Barik

**Affiliations:** 1 Department of Biochemistry, Datta Meghe Medical College, Maharashtra, Nagpur, India; 2 Department of Orthopaedics, All India Institute of Medical Sciences, Maharashtra, Nagpur, India

**Keywords:** Orthopaedic education, non-orthopaedic healthcare professionals, musculoskeletal disorders, continuing medical education (CME), interdisciplinary training, educational strategies

## Abstract

Orthopaedic problems are common in many healthcare settings. This increases the need of non-orthopaedic healthcare professionals (NOHPs) having a basic understanding of musculoskeletal illnesses. Therefore, it is of interest to review the current educational approaches, obstacles and suggestions for enhancing orthopaedic education for NOHPs. This review emphasizes the crucial requirement for comprehensive orthopaedic education for non-orthopaedic healthcare providers to successfully manage the rising global burden of musculoskeletal (MSK) illnesses.

## Background:

Musculoskeletal (MSK) problems are a major global health concern because they can lead to disability, hamper quality of life and greatly increase healthcare expenditures and according to the World Health Organization (WHO), musculoskeletal disorders are a significant contributor to the worldwide burden of disease, leading to diminished mobility, chronic pain and reduced efficiency at work and effective management of these illnesses is essential as part of comprehensive patient care because they are routinely observed in various healthcare settings and comprise a wide spectrum of diseases affecting the muscles, bones, joints and connective tissues [1]. Non-orthopedic healthcare professionals, especially primary care physicians (PCPs), are often the initial point of contact for individuals with MSK issues. They play a critical role in the preliminary diagnosis, initial care and proper referral of various illnesses to guarantee timely and effective treatment [2]. Despite the high frequency of MSK symptoms in primary care and the significant impact these disorders have on patients, there have been identified short comings in the musculoskeletal training provided to non-orthopedic clinicians at different phases of their medical school [3]. Effective management of musculoskeletal problems by non-orthopedic healthcare practitioners has numerous potential benefits for patients and physicians and by differentiating between self-limiting illnesses from those that requires specialized orthopedic therapy; improved orthopedic competence enables PCPs, physician assistants (PAs), nurse practitioners (NPs) and nurses to diagnose a wider range of common MSK problems accurately [4]. This enhanced diagnostic capability reduces unnecessary referrals to orthopedic specialists and develops more appropriate initial management options to optimize healthcare resources and enhance patient access to timely care [5]. Furthermore, non-orthopedic doctors can manage many MSK issues in the primary care setting by having a greater understanding of conservative treatment approaches like exercise therapy, patient education on self-management techniques and the appropriate use of analgesics [2]. As a result, patients may have better results, require less invasive procedures and be more satisfied with their overall experience receiving care [6]. The ability to identify "red flag" symptoms and indicators like infection, cancer, or neurological impairment that may point to serious underlying pathology is emphasized significantly in orthopedic education for non-specialists and furthermore the patient safety and timely access to the right extent of care when needed depend on the ability to recognize these warning signs and quickly refer patients to orthopedic specialists and given the increasing emphasis on inter professional collaboration in healthcare, non-orthopaedic professionals having a basic understanding of orthopaedic principles collaborate and communicate with primary care physicians and specialists, ultimately leading to a more patient-centered and integrated approach to musculoskeletal healthcare [7]. Therefore, it is of interest to explore the impact of orthopaedic education on clinical practice and patient outcomes, the challenges and barriers encountered during its delivery and potential future directions for enhancing the musculoskeletal competency of non-specialist clinicians.

## Current educational approach:

Non-orthopaedic healthcare professionals frequently encounter musculoskeletal (MSK) conditions in clinical practice. To manage these effectively, they require foundational orthopaedic knowledge and clinical skills. Educational strategies have evolved to include formal training, continuing education and resources from professional organizations to bridge this knowledge gap. Professional Organization Resources: Reputable institutions such as the American Academy of Orthopaedic Surgeons (AAOS) [8], the British Orthopaedic Association (BOA) and the AO Foundation [9] provide valuable orthopaedic learning resources. These include: Webinars, Online courses, Articles, publications and Interactive learning modules. These materials cover a broad spectrum of musculoskeletal topics and are designed to benefit both specialists and non-specialists in orthopaedics [3]. Musculoskeletal Education in Medical Training: Musculoskeletal (MSK) conditions are underrepresented in undergraduate medical education, with few dedicated lectures and limited clinical exposure. As a result, medical graduates often lack confidence and adequate knowledge in managing MSK issues. Although postgraduate and residency programs in specialties such as emergency medicine, internal medicine and family medicine typically include MSK training, the scope, duration and quality of this education vary significantly across institutions.

Promising strategies include longitudinal curricula which include integrating MSK topics across training years and dedicated MSK clinics in primary care residency settings [10]. Continuing Medical Education (CME) and Professional Development: For practicing clinicians, continuing education is essential to maintain and enhance orthopaedic knowledge [11]. Available modalities include online modules and courses, Focused workshops and specialized training programs. These educational approaches help clinicians meet specific learning goals and increase their confidence in managing MSK conditions in primary care [12]. Effectiveness of Targeted Orthopaedic Training: Research supports the value of focused orthopaedic education. For example: A one-day undergraduate course improved student knowledge and interest in orthopaedics [13] and A six-week residency program enhanced residents' confidence in surgical skills and clinical readiness [14]. Integrated MSK curricula within residencies resulted in Better physical examination and injection skills and Improved observed performance in MSK assessments [15]. These programs not only strengthen knowledge but also improve practical skills, diagnostic accuracy and confidence in NOHP [14].

## Impact on clinical practice and patient care:

Improved MSK education enhances care quality by supporting accurate diagnosis and effective conservative management [14], enabling early identification of "red flags" such as fever, neurological deficits, night pain, weight loss and trauma with suspected fracture [16]. Patients with potentially significant problems will have timely access to specialized orthopaedic care if non-orthopaedic clinicians are trained to recognize these crucial signs. Educated clinicians can manage common MSK issues like back pain, osteoarthritis, sprains and strains more confidently and effectively. Patient Education Benefits: Orthopaedic training often includes patient education strategies that reduce hospital stays, lower healthcare costs, improve patient function and satisfaction and alleviate anxiety [17]. Interdisciplinary Collaboration: Team-based learning approaches integrate knowledge from orthopaedics, physiotherapy and rehabilitation medicine [18]. Improvements in clinical outcomes have been seen through enhanced communication, clearer role definitions and shared decision-making within healthcare teams due to inter-professional education initiatives in orthopaedics [6].

## Pros and cons of educational modalities:

Various educational modalities offer unique advantages and limitations in musculoskeletal (MSK) training [19]. Online learning is flexible, cost-effective, self-paced and widely accessible, though it may lack opportunities for hands-on skill development [11, 20]. Workshops provide interactive, hands-on training with immediate feedback but are more resource-intensive, requiring faculty time, travel and materials [11, 14]. Simulation-based training offers a safe environment to practice complex skills and enhance decision-making, although it often demands specialized equipment and setup [19]. Blended learning, which combines online content with in-person training, leverages the strengths of both approaches. Effective MSK education should also align with adult learning principles-being self-directed, relevant and focused on practical application [11]. Ultimately, selecting the most suitable educational modality depends on the specific learning objectives, target audience, available resources and logistical constraints.

## Challenges in orthopaedic education for Non-orthopaedic healthcare professionals (NOHPs):

Despite the growing recognition of the importance of orthopaedic education for non-specialists, several persistent barriers limit its effectiveness and reach [20].

## Limited curriculum time:

Medical schools and residency programs often have tightly packed curricula, leaving little room for comprehensive musculoskeletal education [3]. As a result, musculoskeletal topics receive minimal lecture time and clinical exposure and non-orthopaedic specialties may deprioritize orthopaedic content in favor of other high-stakes subjects [21].

## Lack of faculty expertise:

Faculty with sufficient knowledge of musculoskeletal medicine is lacking in many non-orthopaedic disciplines. This can lead to inadequate instruction quality, missed opportunities for interdisciplinary teaching and confidence reduction among educators in delivering musculoskeletal content [10].

## Accessibility issues in underserved areas:

Difficulties faced by clinicians working in underprivileged or rural areas are unique like geographic remoteness limits access to in-person mentorship or training. There may be few or no orthopaedic specialists in the area available for collaboration or consultation or access to online courses may be limited by inadequate internet infrastructure [22].

## Financial and time constraints:

Participation in orthopedic education programs can be affected by high program fees or travel expenses for conferences and workshops. Also, clinical professionals with demanding workloads or on-call responsibilities may find it challenging to fulfil time commitments [21].

## Attitudinal barriers:

Some non-specialists may consider musculoskeletal education as unnecessary, especially if their training is focused on other areas. Some may be reluctant to update long-standing clinical habits. Some may overlook the importance of orthopaedic knowledge in their practice [23]. These difficulties emphasize the necessity of focused, easily accessible and adaptable orthopaedic education approaches that take institutional, logistical and cultural considerations into account. Improving musculoskeletal care in primary and generalist settings requires addressing these obstacles.

## Recommendations for enhancing orthopaedic education for Non-orthopaedic healthcare professionals (NOHPs):

Improving orthopaedic education for NOHPs requires a strategic, multi-level approach that addresses systemic, educational and logistical barriers. Below are key recommendations:

## Curriculum integration and standardization:

To improve musculoskeletal (MSK) education, a longitudinal integration of comprehensive and standardized curricula throughout undergraduate and postgraduate medical training is essential [15]. Repeated exposure to key MSK concepts through sustained and progressively advanced instruction across training years reinforces knowledge retention and skill development. Additionally, providing hands-on experience-through dedicated time for physical examinations, procedural skills such as joint injections and clinical rotations in MSK care-ensures learners develop the practical competencies necessary for effective patient management [24].

## Leveraging technology for accessibility:

To address time and geographic constraints in musculoskeletal (MSK) education, interactive e-learning modules and virtual simulations can be employed through online learning platforms [24]. Blended learning further enhances this approach by combining online education with in-person training, increasing learner engagement while retaining the benefits of hands-on instruction and real-time feedback. These tools offer flexible, accessible learning opportunities without compromising content quality. Additionally, the smartphone applications and telemedicine-based remote learning tools support point-of-care education and ensure continual access to MSK resources, facilitating just-in-time learning in clinical environments [25].

## Faculty development and institutional support:

Enhancing musculoskeletal (MSK) education requires a multifaceted approach that includes investing in the training of non-orthopaedic faculty to confidently deliver MSK content across medical curricula [10]. Resource development is equally important-creating and distributing high-quality; user-friendly educational materials such as visual guides, instructional videos and interactive platforms can significantly enhance learning outcomes [26]. Furthermore, institutional backing through dedicated funding and policy-level support is essential to prioritize MSK education and ensure its sustained integration within medical training programs [27].

## Inter professional and inter departmental collaboration:

It is vital for strengthening musculoskeletal (MSK) education. Fostering partnerships between orthopaedic departments and other specialties-such as family medicine and emergency medicine-can facilitate the sharing of resources and expertise. These collaborations enable the design of interdisciplinary training modules and promote a team-based, collaborative approach to MSK care, ultimately improving educational outcomes and patient management [6].

## Consensus guidelines and core competencies:

Professional bodies have outlined essential musculoskeletal (MSK) competencies for non-orthopaedic healthcare professionals (NOHPs) to ensure effective and comprehensive patient care [16, 28]. These competencies include clinical skills such as focused MSK history-taking, physical examination techniques and the interpretation of basic imaging like X-rays [28]. Procedural skills encompass joint aspirations and injections, as well as immobilization techniques [10]. Management principles cover both non-operative and operative approaches, along with criteria for timely referral to specialists [16]. Additionally, non-technical skills [29] such as effective communication, teamwork and clinical decision-making are critical [29]. On-field emergency care in sports settings, as emphasized in the Team Physician Consensus Statement, is also a key component of MSK competence for practitioners involved in athletic care [30].

## Optimization of referral patterns:

Education-based interventions have a direct impact on improving musculoskeletal (MSK) knowledge among non-orthopaedic healthcare professionals (NOHPs). Enhanced MSK education empowers NOHPs to manage cases confidently within their scope of practice, reduce inappropriate specialist referrals and prioritize timely and appropriate consultations [5]. Conversely, insufficient MSK education has been linked to high rates of unnecessary referrals for conditions that could be effectively managed in primary care settings [31]. Establishing clear referral pathways and reinforcing education can help align referral decisions with best practices [32]. Additionally, patient expectations and systemic limitations within the healthcare system influence referral behaviors and must be acknowledged when designing interventions to optimize care delivery [33].

## Utilization of effective educational modalities:

Accessibility, learner engagement and knowledge retention in musculoskeletal (MSK) education can be significantly enhanced through interactive online modules that offer flexible and self-paced learning. Simulation-based training further supports the development of procedural skills in a safe, controlled environment, allowing for repeated practice and immediate feedback. Blended learning strategies that integrate the strengths of both online and in-person instruction provide a comprehensive educational experience, balancing theoretical knowledge with practical application [19].

## Evaluation and feedback mechanisms:

To ensure ongoing competency in musculoskeletal (MSK) care, it is essential to conduct objective evaluations that assess both knowledge retention and practical skill proficiency. Continuous monitoring through regular feedback mechanisms further supports learner development identifies areas for improvement and reinforces clinical competence over time [34].

## Emphasis on lifelong learning:

Upholding good standards of care in musculoskeletal (MSK) medicine requires fostering a culture of continuous professional development. The non-orthopedic healthcare professionals (NOHPs) should be promoted to remain updated with new research, developing technologies and modernized therapeutic approaches through opportunities for advanced training and continuous continuing medical education (CME). In a rapidly evolving field, this commitment to lifelong learning fosters clinical expertise and adaptability [35].

## Regional adaptation of MSK education in low- and middle-income countries (LMICs):

Training programs should be adapted to the unique circumstances and problems of low- and middle-income countries (LMICs) to increase the accessibility and applicability of musculoskeletal (MSK) education in these areas. This includes creating inexpensive, offline-accessible instructional materials and using peer-to-peer training models to overcome issues with internet connectivity and the availability of specialists. Training materials should be available in local languages and include case studies unique to a region to guarantee cultural and linguistic appropriateness. To improve policy integration and optimize impact across health systems, MSK education initiatives should also be carefully associated with more general national health targets, such as the prevention of non-communicable diseases and the goals of universal health coverage [36, 37- 38].

## Prospects for research and innovation in the future:

To understand how different educational approaches affect patient outcomes, medical expenses and clinical effectiveness in musculoskeletal (MSK) care, impact studies should be carried out. In addition, investigating the long-term retention of MSK information is essential since it aids in determining the best practices for skill reinforcement. To provide equal access to high-quality MSK education and care, it is also crucial to create scalable models that can be modified for use in various practical contexts, such as underprivileged and resource-constrained locations [39]. Clinical decision-making and adherence to best practices can be enhanced by incorporating MSK education into electronic medical records (EMRs) through interactive prompts or AI-based decision-support technologies. Additionally, virtual reality (VR) simulations and gamified learning platforms can improve student engagement and make it easier for them to master intricate assessment and procedural skills in a risk-free, immersive setting. The different functions of NOHPs, including community health workers, sports medicine clinicians and primary care physicians, can be accommodated by implementing a tiered competency structure that offers modular training tracks that correspond with their particular clinical responsibilities. When executed collectively, these strategies promote sustainable, context-sensitive and scalable advancements in MSK care delivery [40-41].

## Interpretation, evaluation and feedback [42]:

Standardized assessment criteria must be incorporated into the planning, execution and outcome monitoring of musculoskeletal (MSK) training programs for non-orthopaedic healthcare professionals (NOHPs) to regulate educational policy and promote institutional reforms in this area. The measurable domains listed below are suggested:

[1] Knowledge and skill acquisition: Multiple-choice questions (MCQs) and checklists for the Objective Structured Clinical Examination (OSCE) should be used to evaluate knowledge and skills before and after an intervention. MSK assessment methods and procedural competencies should be the main emphasis of OSCEs. To ascertain long-term effectiveness, retention of knowledge and skills should be assessed three, six and twelve months after training.

[2] Clinical confidence and practice change: Likert-scale questionnaires before and after the training can be utilized to evaluate self-reported confidence levels. Improvements in clinical practice can be monitored by audits of MSK case management, which include the frequency of independent case management by NOHPs and the decrease in unnecessary referrals to specialists. Analytics from electronic medical records (EMRs) can be utilized to routinely track these patterns.

[3] Patient-centered outcomes: After MSK consultations, standardized feedback forms should be utilized for assessing patient satisfaction. Additional indicators can include the amount of time required to obtain a suitable referral or management resolution. Validated methods like the Quick Disabilities of the Arm, Shoulder and Hand (QuickDASH) for upper limb function and the Visual Analog Scale (VAS) for pain can quantify functional results.

[4] Institutional and system-level impact: By examining participation rates, broken down by geographic location, clinical specialty and healthcare environment (rural versus urban), training programs' reach should be monitored. A cost-benefit analysis should be carried out to compare the costs of arranging the training program with the financial savings due to decreased referrals, diagnostic imaging and other downstream healthcare charges. The number of instructors trained and the total number of instructional hours delivered should be tracked to track faculty development.

[5] Equity and accessibility: It is important to assess the geographic distribution and accessibility of educational programs, especially in contexts with low resources and underserved populations. Program reach should be evaluated by measuring utilization rates in these locations. Training resources' adaptability must be reviewed as well to make sure they satisfy language, cultural and technological requirements.

[6] By implementing these evidence-based recommendations, institutions can improve outcomes across healthcare systems by empowering non-orthopaedic practitioners to provide patient-centered, efficient and effective musculoskeletal care.

## Conclusion:

The need for comprehensive orthopedic education for non-orthopedic healthcare providers to effectively address the growing global burden of musculoskeletal (MSK) conditions is described. Targeted educational interventions have been shown to enhance the knowledge, skills, and confidence of doctors, resulting in better patient outcomes and more efficient healthcare resource utilization. To maximize the impact of these programs, challenges related to curriculum duration, faculty expertise, and accessibility, especially in resource-limited settings, must be tackled, and evaluation metrics should be integrated into policy and planning frameworks to promote sustainable improvements.

## Source of funding:

None.

## Patient consent:

Since this is a review, consent to participate were not required for this study.

## Ethical clearance:

Not applicable.

## Declaration:

This article is not under consideration in any journal and its preprint is not published anywhere.
